# Findings of a Four-Year Randomized Controlled Clinical Trial Comparing Two-Piece and One-Piece Zirconia Abutments Supporting Single Prosthetic Restorations in Maxillary Anterior Region

**DOI:** 10.1155/2016/8767845

**Published:** 2016-01-04

**Authors:** Guerino Paolantoni, Gaetano Marenzi, Andrea Blasi, Jolanda Mignogna, Gilberto Sammartino

**Affiliations:** ^1^Private Practice, 80131 Naples, Italy; ^2^Division of Oral Surgery and Implantology, Department of Neurosciences, Reproductive and Odontostomatological Sciences, University of Naples Federico II, Via Pansini 5, 80131 Naples, Italy; ^3^Department of Neurosciences, Reproductive and Odontostomatological Sciences, University of Naples Federico II, Via Pansini 5, 80131 Naples, Italy

## Abstract

The purpose of this randomized controlled study is to investigate the clinical results obtained over four years and incidence of complications associated with* one*- versus* two*-piece custom made zirconia anchorages, in single tooth implant-supported restorations of the maxillary anterior region. Sixty-five patients, with a total of 74 missing maxillary teeth, were selected in the period from February 2007 to July 2010. Two different ways of custom made zirconia abutment and final prosthetic restoration were evaluated: a standard zirconia abutment associated with a pressed layer of lithium disilicate with an all-ceramic cemented restoration versus one-piece restoration with the facing porcelain fired and pressed straight to the custom made zirconia abutment. In 29 cases, the restoration consisted of an all-ceramic restoration for cementation (two pieces); in 45 cases the restoration was a screw-retained restoration (one piece). Three all-ceramic restorations broke during the observation time. Two one-piece restorations fractured after 26 months. At follow-up examination there were no significant differences between one-piece and two-piece groups regarding the PI, BI, and MBL. Awaiting studies with longer follow-up times, a careful conclusion is that zirconia anchorages for single-implant restorations seem to demonstrate good short-term technical and biological results.

## 1. Introduction

The clinical advantages of ceramic abutment led to a development of their use in implant-supported restorations [1]. Among ceramics, zirconia (ZrO_2_) has probably been the most widely studied material in the last twenty years following the discovery of the stress-assisted tetragonal-to-monoclinic transformation in partially stabilized zirconia alloys [2]. This phase transformation, usually referred to as martensitic transformation, is accompanied by a 3–5% volume expansion, which helps to arrest (or at least to minimize) the propagation of cracks [2]. Many authors evidenced good short-term technical, biological, and aesthetic results [3] in the use of zirconia abutments for single-implant restorations and these clinical results have been reported for different implant systems [4, 5]. The introduction of individually prepared abutments improved functional and aesthetic outcomes. The custom made abutment, according to the individual anatomic needs, allowed a personalised placement of the margin of the prosthetic restoration ensuring satisfactory aesthetic results [6]. In the aesthetic zone, a customized zirconia abutment can accommodate a nonideal implant position while simultaneously supporting the morphological features of the soft tissues and overlying restoration [6].

The aim of this randomized controlled study is to investigate the clinical results obtained over four years and incidence of complications associated with* one*- versus* two*-piece custom made zirconia abutments, in single tooth implant-supported restorations of the maxillary anterior region.

## 2. Material and Methods

### 2.1. Study Population

Sixty-five patients, including 44 females and 21 males, with a total of 74 missing maxillary teeth, mean age 53 ± 4 years, were selected in the period from February 2007 to July 2010, at the Department of Neurosciences, Reproduction and Odontostomatological Sciences of the University of Naples “Federico II.” The study was designed as a controlled randomized trial on patients who needed single-implant restoration in the maxillary anterior area with healthy nonrestored neighboring teeth. Two different ways of custom made zirconia abutment and final prosthetic restoration were evaluated: a standard zirconia anchorage (ART, Thommen Medical AG, Grenchen, Switzerland) associated with a pressed layer of lithium disilicate with an all-ceramic cemented restoration (two pieces) versus one-piece restoration with the facing porcelain fired and pressed straight to the custom made zirconia anchorage (ART Anchorage, Thommen Medical AG, Grenchen, Switzerland). The prosthetic treatment of the patients was chosen with coin toss randomization [7]. This study was conducted in accordance with the 1975 Helsinki requirements then revised in 2008. The selected patients were nonsmokers; they were verbally informed and gave their written consent. The following patients were excluded from the study: patients refusing to sign the agreement, patients with poor oral hygiene (Full-Mouth Plaque Score (FMPS) ≥ 20% at baseline [8]; Full-Mouth Bleeding Score (FMBS) ≥ 20% at baseline [8]) or with a periodontal disease, and patients with local infections of the soft tissues or affected by psychiatric disorder or pregnant women.

### 2.2. Surgical Procedure

**Figure 1:**
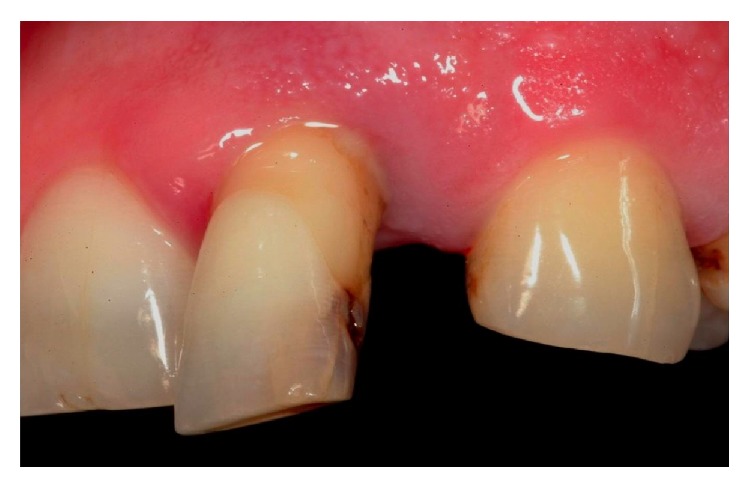
Preoperative view of left lateral incisor affected by periodontal disease.

**Figure 2 fig2:**
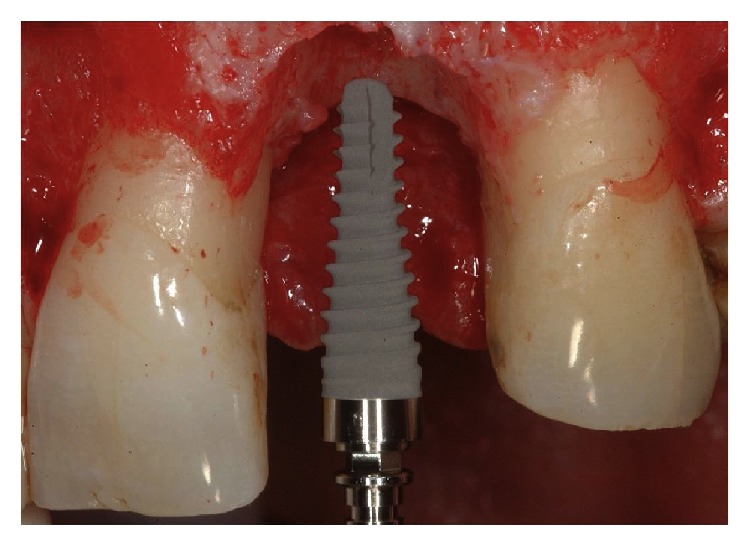
Delayed placement of single tooth implant 30 days after tooth extraction.

**Figure 3 fig3:**
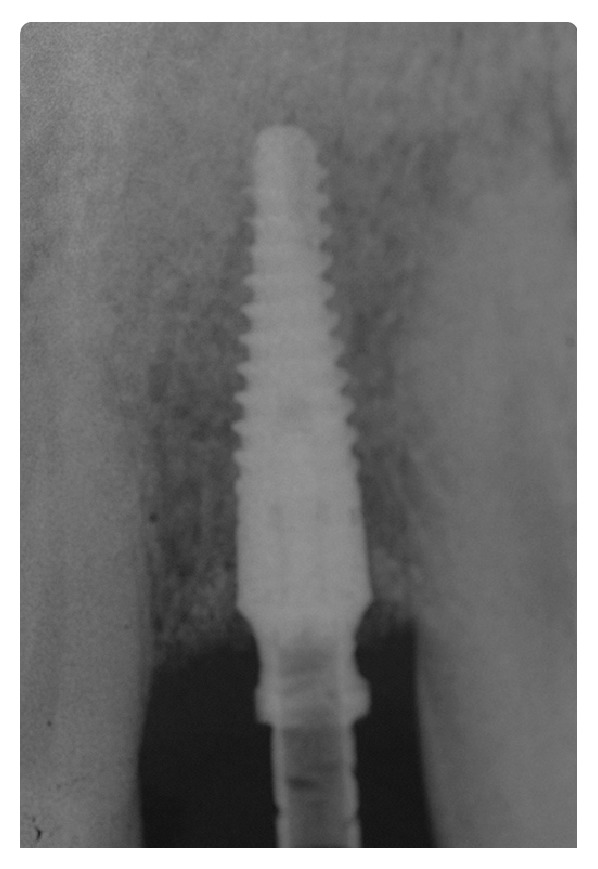
Periapical Rx immediately after implant placement with provisional screw anchorage.

**Figure 4:**
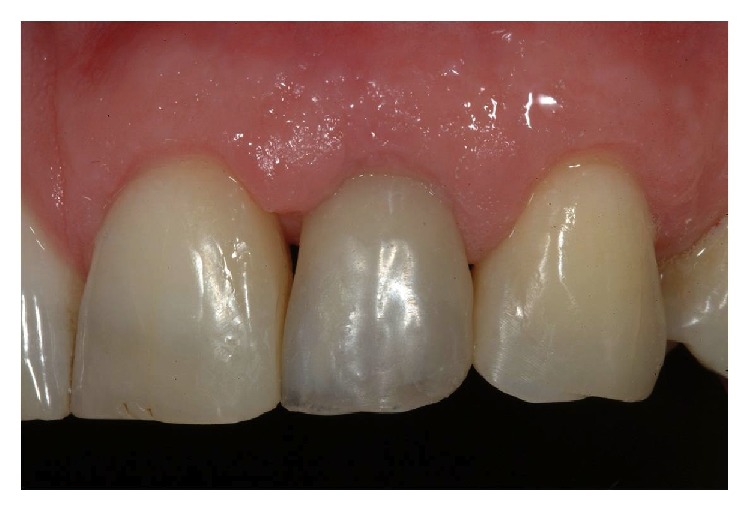
Temporary screw rehabilitation.

An alveolar nerve block infiltration was administrated with local anesthesia using 2% mepivacaine; 51 implants ((Thommen Medical AG, Grenchen, Switzerland) 68.9%) were placed with a one-stage procedure [9] (Figures 1 [Fig fig2] [Fig fig3]–4) and 23 implants (31.1%) were placed with two-stage procedure [10] in the maxillary anterior region (from right canine to left canine).

Each implant placement was performed with the aim of finding an ideal three-dimensional implant position for better aesthetic results: the respect of the vestibular bone crest, the distance of the implant body not exceeding 1.5 mm from the adjacent root surface, and the position of the implant shoulder approximately 2 mm apical to the midfacial gingival edge of the planned restoration. 26 patients were submitted to GBR in the immediate postextraction time to allow an implant placement following the previous criteria.

### 2.3. Prosthodontic Procedure

In all cases the prosthetic procedures were performed six months after implant placement. Two different ways of custom made zirconia anchorage and final prosthetic restoration were evaluated: a standard zirconia anchorage (ART, Thommen Medical AG Waldenburg, Switzerland) associated with a pressed layer of lithium disilicate with an all-ceramic cemented restoration (two pieces) versus a one-piece restoration with the facing porcelain fired and pressed straight to the custom made zirconia anchorage (ART Anchorage, Thommen Medical AG Waldenburg, Switzerland). In two-piece group the ceramic restorations (lithium disilicate Ivoclar-Vivadent) were cemented with resin cement (3M, Relyx Unicem 2 Automix).

All zirconia abutments, once a clinical check is made, were tightened with 25 N/cm according to manufacturer's instruction (Thommen Medical AG, Grenchen, Switzerland) and then we proceeded to the cementation.

Radiographs taken at the time of prosthesis delivery (i.e., baseline) and at the follow-up examination were used to calculate the radiographic bone level at the mesial and at the distal sites of the implants. The radiographic linear distance from the implant shoulder to the first bone-to-implant contact was used to calculate the marginal bone levels. All the radiographs were taken by applying the longcone technique and by using a film holder [11]. The films were digitized and the location of the marginal bone level in relation to the implant shoulder was assessed at the mesial and the distal aspect by using a software program (VixWin Platinum, KaVo Dental GmbH, Biberach, Germany). To take into account the anatomic magnification and distortion in the films, the linear dimensions of the digitized images were calibrated. This was achieved by setting the scale in the image to the known distance between two implant threads (e.g., 1.25 mm) [12].

The single tooth implant-supported restorations require a minimum 4-year follow-up period (57–83 months).

The examination comprised registration of technical and biological data at 12, 24, 36, and 48 months after the prosthesis insertion time.

The technical recordings comprised fractures of the abutment, restoration and facing porcelain, loss of retention of the abutment (anchorage screw loosening), or restoration.

The biological parameters included mobility of the implant (yes or no), Plaque Index (PI) [13], Bleeding Index (BI), and the marginal bone loss (MBL). The examiner assessed the probing depth (PD) inserting UNC-15 periodontal probe (Hu-Friedy, Chicago IL, USA) parallel to the root surface and directed apically toward the perceived location of the apex of the root with a force of 30 g until slight resistance was felt.

## 3. Results

The causes of missing teeth were endodontic failure (28 cases; 37.8%), residual roots (17 cases; 22.9%), nonrestorable caries (9 cases; 12.2%), and agenesis (20 cases; 27.1%).

**Figure 5:**
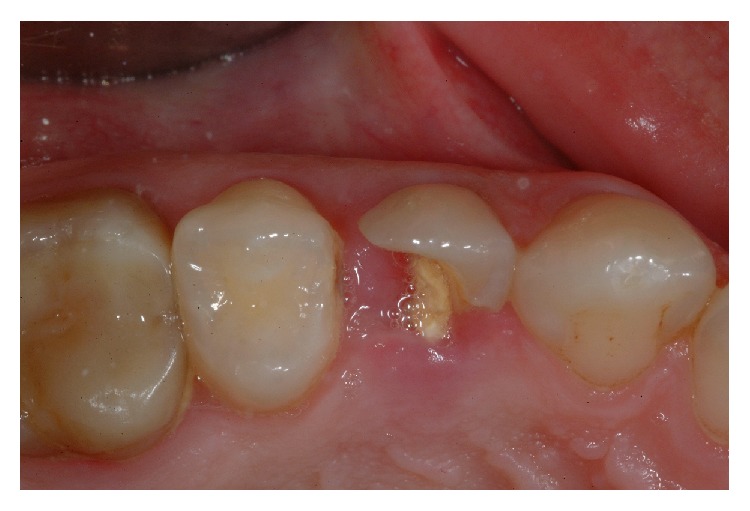
Preoperative occlusal view of the maxillary first right premolar.

**Figure 6 fig6:**
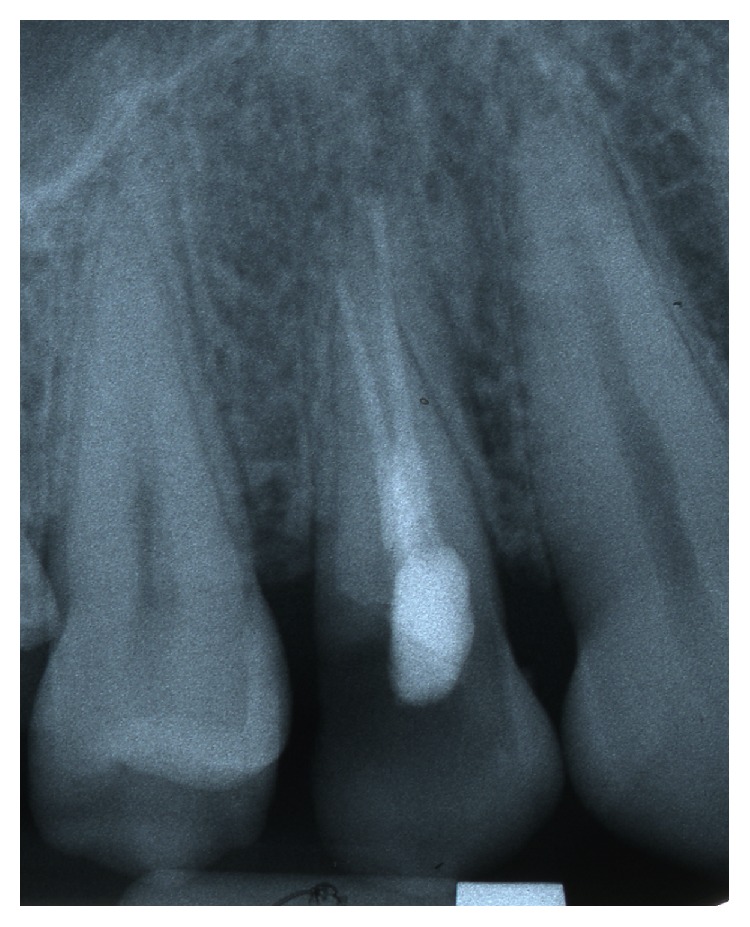
Periapical X-ray at baseline showing the tooth fracture and decay of the first right premolar.

**Figure 7 fig7:**
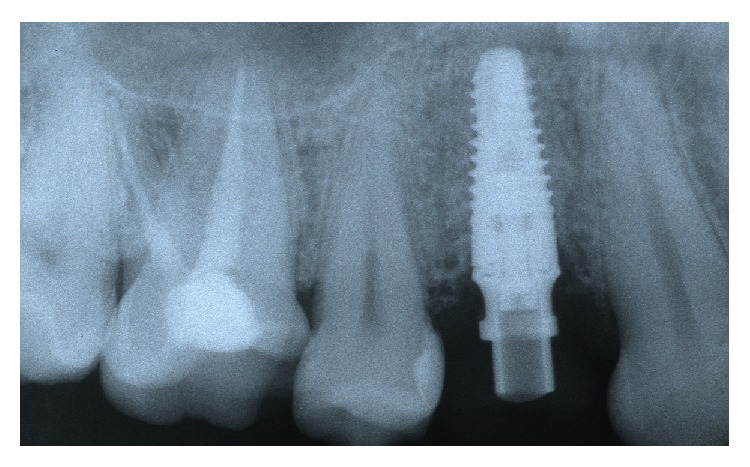
Periapical Rx of temporary screw rehabilitation.

**Figure 8:**
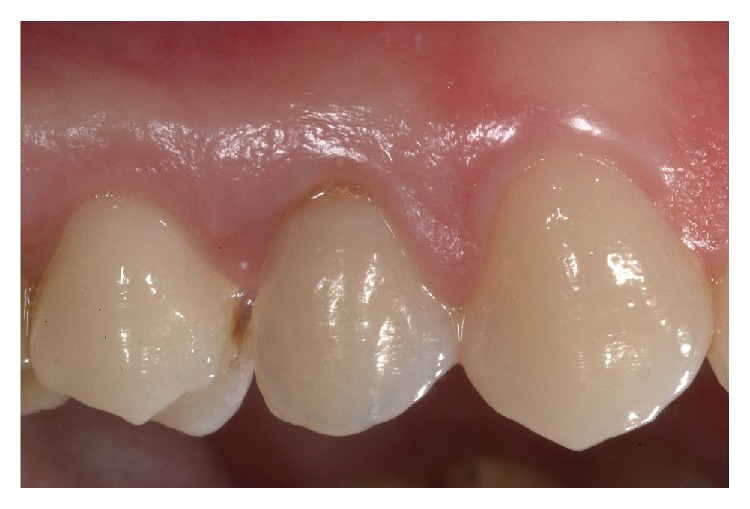
The definitive custom zirconia-disilicate pressed anchorage and disilicate monolithic restoration.

65 patients received one implant, but 9 needed two single nonadjacent prosthetic restorations. 51 fixtures (68.9%) were placed with a one-stage procedure [9] with a healing period of 12 months (Figures 1–4); 23 fixtures (31.1%) were inserted with a two-stage procedure [10] and a healing period of 6 months (Figures 5 [Fig fig6] [Fig fig7]–8). GBR was required for 26 implants (8 in one-stage group and 18 in two-stage group; in these cases the healing period was 12 months). The implant sites and dimensions are summarized in Table 1. The number of the different fixture dimensions used as well as their prosthetic rehabilitation is reported in Table 2.

**Figure 9:**
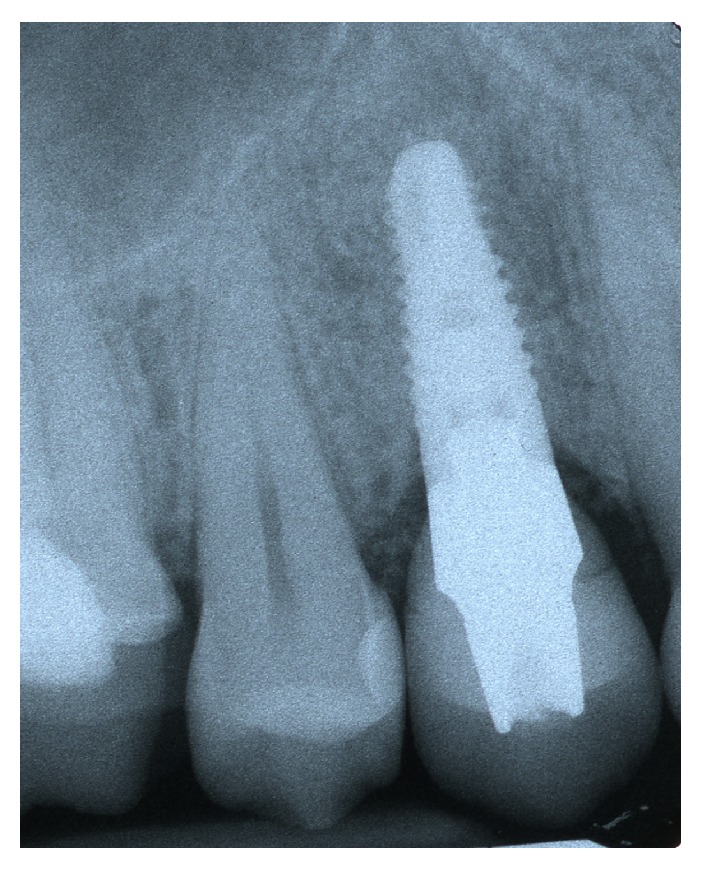
The periapical radiograph of the implant and the cement restoration (two pieces) 36 months after implant loading. Almost no edgeal bone loss was noted around the implant.

**Figure 10:**
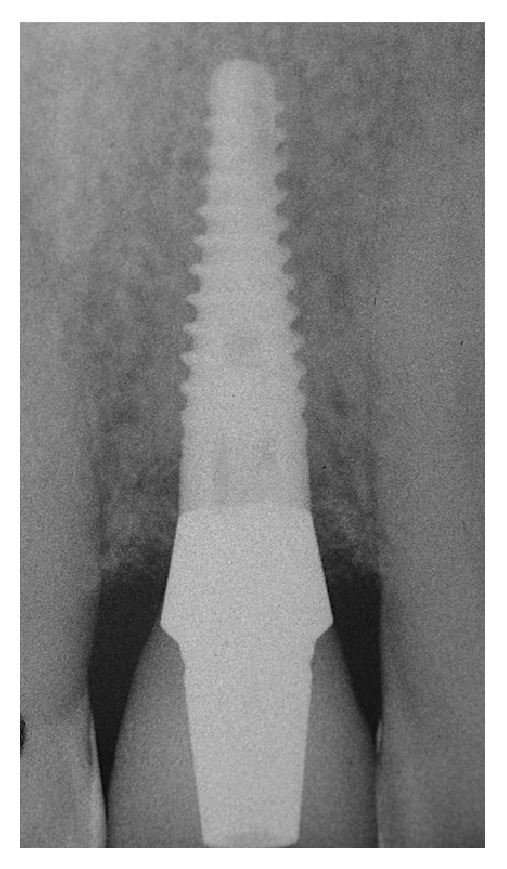
The periapical radiograph of the implant and the screw-retained restoration (one piece) 38 months after implant loading. Almost no edgeal bone loss was noted around the implant.

All patients showed good compliance and the healing period was uneventful. At 4 years of follow-up no patients suffered from pain or peri-implant infection; the implant survival rate was 100% and none of the implants showed mobility. In 29 cases, the restoration consisted of an all-ceramic restoration for cementation (two pieces) (Figure 9); in 45 cases the restoration was a screw-retained restoration (one piece) (Figure 10).

Three (10.3%) all-ceramic restorations broke during the observation time. All two-piece restorations, except one (3.4%), had excellent edge fit between restoration and abutment.

Only 2 (4.4%) one-piece restorations fractured after 26 months. No anchorage screw loosening was reported. The mean PI and rehabilitation BI values at 12, 24, 36, and 48 months after the prostheses insertion time are reported in Table 3.

Kaplan-Meier's survival analysis was performed to compare the two groups. The implant placement technique (one stage or two stages) did not significantly influence the clinical outcomes. At follow-up examination there were no significant differences between one-piece and two-piece groups regarding the PI, BI, and MBL (Tables 3 and 4).

**Figure 11:**
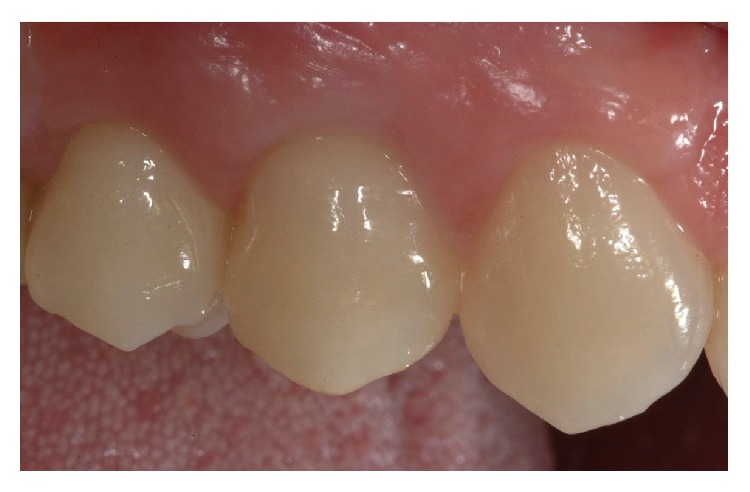
Intraoral view of the final rehabilitation after 12 months.

**Figure 12:**
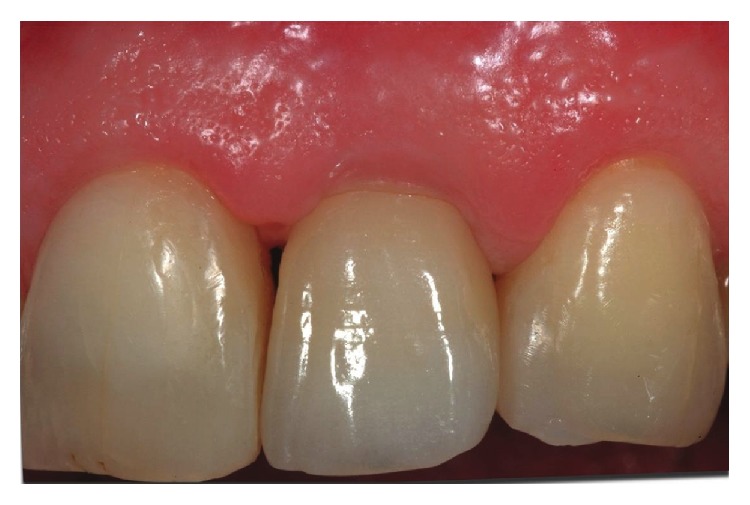
Buccal view of the final rehabilitation after 15 months.

Only 2 implants (6.8%) had lost more than tone thread (Figures 11 and 12). Both these implants supported a two-piece restoration and the quality of the peri-implant keratinized mucosa was able to cover the restoration-anchorage connection assuring a good aesthetic result.

## 4. Discussion

The zirconia (zirconium dioxide) characteristics have led to its increased use for many dental applications [14]. Many advantages of ceramic in comparison with metal anchorages have been reported: less mucosal discoloration [15], less adhesion of bacteria [16], low or no cytotoxicity [17], and a mucosal attachment very similar to titanium one [18]. Zirconia anchorages for single-implant restorations seem to demonstrate good short-term technical, biological, and optical esthetic results with different implant system [9, 10, 14]. Clinical studies on single-tooth implants evidenced that zirconia abutments, supporting single-tooth implant restorations in the anterior and premolar regions, showed a survival rate of 100% over 3-4 years [19, 20]. Moreover the low prosthetic restoration fracture events reported showed the zirconia abutment ability to tolerate the occlusal forces of the aesthetic maxillary region. Although the clinical advantages of zirconia anchorages are very promising, further investigations are needed.

A 3-year randomized controlled clinical trial comparing customized zirconia and titanium anchorages showed no difference in the outcome from technical, biological, but also aesthetical points of view [21]. Another recent review showed no difference in mucosal discoloration between zirconia and titanium anchorages, contradicting the previous studies [22, 23].

The loosening of the anchorage screw did not occur in the present study confirming some recent articles performed either in vivo [14–16] or in vitro [1, 2] which reported screw loosening as a rare event in single-implant restorations. Screw loosening depends on the precision and the extension of the contact area between the retaining screw and the anchorage; in the clinical cases reported, the used implants system connections seem able to tolerate the normal occlusal forces of the anterior maxillary teeth.

Our study reports the clinical, radiographic outcomes of two different prosthetic ways to use the custom made zirconia anchorages and evidence that the custom zirconia anchorages used for one- and two-piece restoration restorations performed well over a follow-up period of up to 4 years with satisfaction by the patients.

However, there were few complications during our observation time. Two one-piece restorations with the facing porcelain fired straight to the abutment fractured after 26 months. One of this fractures could be related to functional overload due to the loss of a mobile prosthesis restoring posterior teeth. This abutment was replaced and no further problems occurred. Although in the two-piece restoration 3 fractures are reported, data from the present study show no statistically significant differences between one-piece and two-piece prostheses in terms of fractures and other complications. At 48 months' follow-up there were no statistical differences either in the peri-implant edgeal bone loss or in soft-tissue conditions between the screw-retained one-piece restorations and single-tooth implant restorations cemented to the anchorage (Tables 3 and 4).

About the MBL the limitations of the radiographic technique and the method of measurement should be considered.

The survival rate and the rate of technical and biological complications in the present study were similar to the rates reported in a systematic review of ceramic anchorages by Sailer et al. [24]. The present study adds only a small number of zirconia abutments and the observation time is still limited. With respect to the laboratory findings that physical properties of zirconia like those of other ceramics may deteriorate with aging [25], it is safe not to extend the present results straight to long-term outcome of zirconia anchorages.

## 5. Conclusions

Awaiting studies with longer follow-up times, a careful conclusion is that zirconia anchorages for single-implant restorations seem to demonstrate good short-term technical and biological results.

## Figures and Tables

**Table 1:** 

Missing teeth	Number and implant dimensions
Left central incisor	12 (4.00 mm–9.5 mm) 5 (4.00 mm–11 mm)

Right central incisor	11 (4.00 mm–9.5 mm) 8 (4.00 mm–11 mm)

Left lateral incisor	4 (3.3 mm–9.5 mm) 4 (3.3 mm–9.5 mm) 4 (4.00 mm–9.5 mm) 2 (4.00 mm–11 mm)

Right lateral incisor	5 (3.3 mm–9.5 mm) 2 (3.3 mm–9.5 mm) 6 (4.00 mm–9.5 mm) 3 (4.00 mm–11 mm)

Left canine	3 (4.5 mm–11 mm) 1 (4.5 mm–9.5 mm)

Right canine	2 (4.5 mm–11 mm) 2 (4.5 mm–9.5 mm)

**Table 2:** 

Number of implants	Implant dimensions	One-piece restorations	Two-piece restorations
33	4.00 mm–9.5 mm	21	12
18	4.00 mm–11 mm	10	8
9	3.3 mm–9.5 mm	4	5
6	3.3 mm–11 mm	4	2
3	4.5 mm–9.5 mm	2	1
5	4.5 mm–11 mm	4	1

**Table 3:** 

Follow-up 48 months	Plaque Index	Bleeding Index
One-piece restoration	0.47 ± 0.50	0.47 ± 0.50
Two-piece restoration	0.48 ± 0.51	0.24 ± 0.44
Significance	0.893	0.053

**Table 4:** 

Follow-up 48 months	Edgeal bone loss
One-piece restoration	1.4 ± 0.99
Two-piece restoration	1.17 ± 0.89
Significance	0.342
